# Magnetomotility of untethered helical soft robots†

**DOI:** 10.1039/c9ra01775e

**Published:** 2019-04-10

**Authors:** Jeong Eun Park, Jisoo Jeon, Jae Han Cho, Sukyoung Won, Hyoung-Joon Jin, Kwang Hee Lee, Jeong Jae Wie

**Affiliations:** Department of Polymer Science and Engineering, Inha University Republic of Korea wie@inha.ac.kr; World Class Smart Laboratory (WCSL), Inha University Republic of Korea

## Abstract

Magnetically active helical soft robots were synthesized to achieve tether-less manipulation of the magnetomotility in order to avoid the on-board weight penalty and the distance restrictions originating from connection lines. Magnetic iron particles were dispersed in elastomeric polymer matrices and pre-cured in a two-dimensional film geometry, followed by post-curing in a three-dimensional (3D) helical geometry. To manipulate movements of the 3D helical soft robots, an external magnetic field was applied by placing a neodymium permanent magnet on a motorized linear translation stage. The 3D helical geometry of the soft robots enabled efficient maneuvering with local deformations and a low magnetic threshold for actuation by the introduction of the rolling resistance unlike the absence of the local deformations observed for rigid 3D coils. As rolling is induced by the action and reaction with the substrate, the helix angle causes divergence of the soft robots from linear translational motility. In order to regulate the directionality of rolling and to minimize temporal and spatial deviation of the soft robots, the magnitude of the magnetic flux density and the velocity of the permanent magnet on the linear stage were investigated.

## Abstract 2

Magnetically active helical soft robots were synthesized to achieve efficient tether-less manipulation of the magnetomotility in order to avoid the on-board weight penalty and the distance restrictions originating from connection lines.

## Graphical abstract

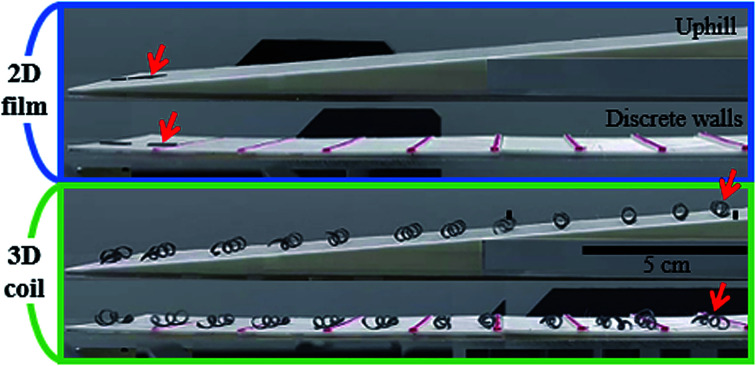

## Introduction

The simplicity of the design of soft robotic systems has fueled interest as a means of achieving locomotion without electrical circuits. Furthermore, curvature can easily be achieved in soft robots with a joint-less design, and the low mechanical stiffness of these devices is appropriate for safe operation in biomedical applications.^1^ Since the first report of a pneumatically actuated elastomer, various soft robotic systems have been reported, including caterpillar locomotive soft robots,^2^ artificial tentacles,^3^ elastomeric walkers,^4^ and octobots.^5^ However, most tethered pneumatic soft robots suffer from a confined working distance and entanglement of the connection lines, limiting their motional complexity.

As an example of untethered soft robotic systems, helical soft robots responsive to photomechanical manipulation were developed by incorporating light-active molecular machines within liquid crystalline polymer networks.^6^ However, the photoactive system suffered from an accompanying temperature increase due to photothermal effects, prohibiting thermo-sensitive applications. Spatially closed systems are also not favorable for photomechanical actuation due to the unsecured light propagation pathway, particularly for short wavelength light. Even with the use of transmissive long wavelength light, the issues of limited penetration depth and low photon energy levels restrict effective photomechanical actuation. In addition, the large amount of photon energy absorbed by the photoactive molecular machines within soft robots can promote degradation of the constituent polymers upon long-term irradiation.

To achieve untethered manipulation of soft robots under ambient conditions, a magnetic field can be employed as an external stimulus. For example, the magnetically-driven swimming motion of microrobots was reported for magnetite-coated bacteria^7^ and hydrogel-magnetite composites.^8^ In addition, the dynamic movements of soft robots has been reported, including multimodal actuation^9^ and the origami-inspired 2D-to-3D transformation of Ecoflex–NdFeB composites.^10^ In this study, we synthesize 3D helical polydimethylsiloxane (PDMS)–iron particle composites for the magnetically-driven regulation of soft robots. The helical geometry of the soft robots is advantageous for achieving efficient motility as the helix introduces rolling resistance instead of sliding resistance, as well as reduced weight in comparison to that of the cylindrical geometry. To date, most helical magnetic robots have been fabricated with multilayers of metallic materials (*i.e.*, a Ni/Ti bilayer, InGaAs/GaAs/Cr trilayer, or Cr/Ni/Au trilayer), and focus has been placed on their magnetic swimming in viscous sustaining fluidic media.^11^ During the swimming motion, the direction of the translational motility is perpendicular to the rotational direction of the helix, like screw-motions. In this study, we report magnetic regulation of macroscopic helical soft robots on substrates, where uniaxial displacement of a permanent magnet underneath the soft robots induces translational motility with directionality along the rotational axis. Ferromagnetic iron particles are randomly dispersed in PDMS elastomers and the composite films are processed into a 3D helical geometry *via* a two-step polymerization method. The dispersion of the iron particles is visualized by SEM micrographs and quantitatively evaluated by local connected fractal dimension analysis. To understand the fundamentals underlying the dispersion of the heavy metal particles, the terminal velocity of the iron particles is calculated.

The efficient maneuver of 3D helical soft robots is contrasted with that of a 2D flat film on up-hill as well as discrete step obstacles. As the rolling motility of the helical soft robots is operative only when the magnetically induced momentum exceeds the rolling resistance, the components of the magnetic flux density are deconvoluted and it is demonstrated that the magnetic component along the translational moving axis is the crucial factor governing the rolling motility, rather than the magnitude of the total magnetic flux density. Despite the various aforementioned advantages, the helical geometry obstructs linear translational motility originating from the helix angle. To achieve linear translational motility of helical soft robots, the magnetic flux density and velocity of the magnet are optimized to minimize the temporal and spatial deviation of the soft robots from the targeted position. Controlled linear and non-linear translational motility of 3D helical soft robots will provide a pathway-adaptive maneuvering strategy that is operative at low magnetic flux density.

## Experimental

The polydimethylsiloxane (PDMS) and iron particle (BASF, CIP CC grade) composites were prepared by a two-step polymerization method. First, the Sylgard 184 PDMS precursor (Dow Corning) and a crosslinking agent were mechanically mixed in a 10 : 1 ratio with the inclusion of iron particles at 3 vol% loading. The PDMS precursor–iron particle mixture was degassed in a vacuum chamber and then pressed into a 0.4 mm thick film. Here, a compliant aluminum foil was employed for facile molding of the film into the helical geometry. The mixture was pressed at 600 psi for 10 s and pre-cured at 80 °C for 5 min. The pre-cured film was cut into strips with dimensions of 67 mm × 1.5 mm × 0.4 mm (length × width × thickness). The pre-cured strip was coiled (helical diameter of 3.7 mm and helix angle of 45°) and fully cured at 80 °C for 1 h. The maneuver of the helical soft robots was by applying a neodymium permanent magnet (NdFeB, Kingkong Magnet, N35 grade). The magnet was placed beneath the substrate on a motorized linear translation stage. The distance between the magnet and the soft robots determines the magnitude of the magnetic flux density. The variation of the magnetic flux density according to the magnet-to-sample displacement was calculated by the ANSYS Discovery AIM R19.2 program using the initial measured magnetic flux density. In this study, four different initial magnetic flux densities were employed (0.05, 0.07, 0.10, and 0.15 T). The magnet on the linear translation stage was displaced at four different velocities (5, 10, 20, and 50 mm s^−1^).

## Results and discussion

**Fig. 1:**
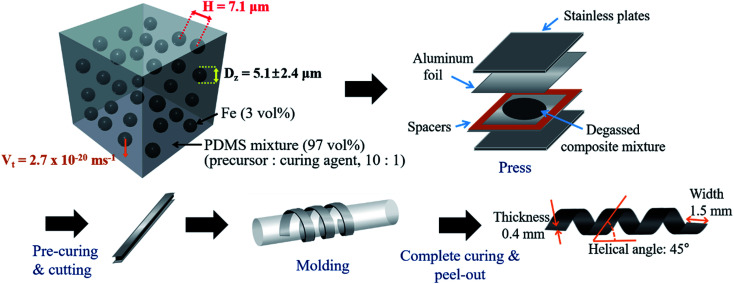
Scheme for preparation of magnetically active helical soft robots *via* two-step polymerization method.

**Fig. 2:**
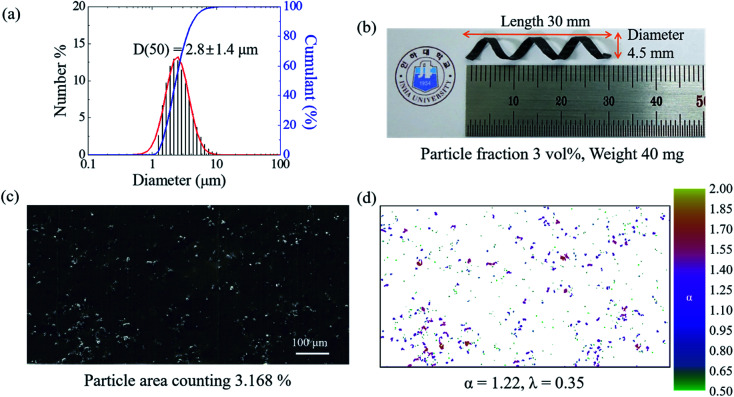
Analysis of magnetic iron particles and PDMS composites. (a) Size distribution of iron particles; the black histograms are the number intensity for the diameter of the iron particles; the blue line is the cumulant value of the intensities; the red line is the lognormal fitting of the particle size distribution. (b) Optical image of magnetically active helical soft robot. (c) Cross-section SEM micrograph of the soft robots. (d) Local connected fractal dimensional analysis from the binary image in (c).

As shown in Fig. 1, the magnetically active helical soft robots were synthesized by a two-step polymerization process. The PDMS–iron particle mixture was degassed and then pre-cured at 80 °C for 5 min in a 2-D film geometry. The pre-cured film was coiled into a 3-D helix and fully cured at 80 °C for 1 h. The number-averaged diameter of the iron particles was 2.8 ± 1.4 μm, as measured by using a particle size analyzer (PSA) and fitting to the asymmetric lognormal distribution, wherein the smaller particles outnumbered the larger particles (Fig. 2a).^12^1
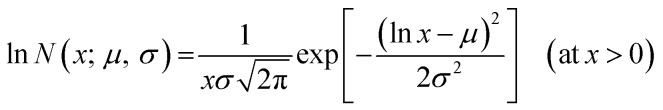


In eqn (1), *μ* is the mean value and *σ* is the standard deviation of the variable's natural logarithm. Here, *μ* is the location parameter, which indicates the location on the *x*-axis, and *σ* is the shape parameter, which reflects the general geometry of the distribution.^13,14^

The *z*-averaged diameter, *d*_*z*_, of the iron particle was calculated to be 5.1 ± 2.4 μm on the basis of the PSA results by applying eqn (2).2
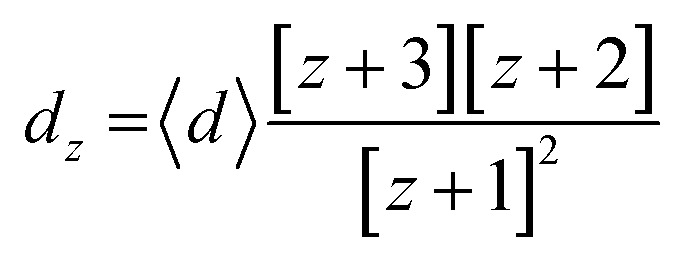
3
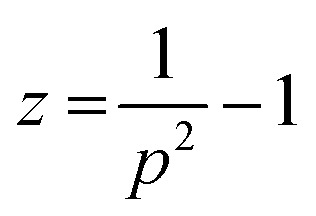
4
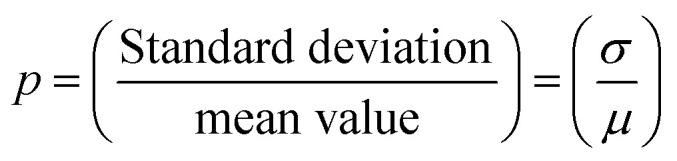
Here, 〈*d*〉 is the mean diameter and *p* is the polydispersity index, corresponding to the standard deviation, *σ*, divided by the mean value of the number-averaged diameter of the iron particles. The *z*-averaged diameter was applied, as the *z*-average is more suitable for quantifying the scattered intensity of the particles and is insensitive to noise.^15,16^ Further, the *z*-averaged diameter was employed to place more weight on the larger particles, as the larger particles have a higher sedimentation rate, which is undesirable for achieving a uniform particle distribution in the composites.

The polymer matrix was investigated. The density of the magnetic iron particles used herein was 7.9 g cm^−3^, which can lead to a substantial sedimentation force under the influence of gravity. In addition to the gravitational force (*F*_g_), the iron particle experiences a drag force (*F*_d_) and buoyant force (*F*_b_) in the highly viscous sustaining fluid, PDMS. The drag force for highly viscous fluids with a very low Reynolds' number can be described by the Stokes' drag.^17^

From the force balance equation (*F*_d_ + *F*_b_ = *F*_g_), the terminal velocity, *v*_t_, can be calculated according to Stokes' law:^18^5
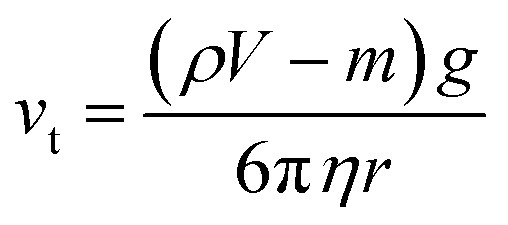
where *ρ* is the density of the PDMS fluid, *V* is the total volume, *m* is the particle mass, *g* is the gravitational constant, *η* is the viscosity of the sustaining fluid, and *r* is the particle radius. From the iron particle size investigated by PSA analysis, the terminal velocity of a 5.1 μm iron particle in PDMS is calculated to be 2.7 × 10^−20^ m s^−1^ (see ESI, S1 and S2†). Given the small value of the terminal velocity, negligible sedimentation was expected. Notably, the viscosity of PDMS will increase significantly during the thermal curing process, thereby further prohibiting sedimentation of the iron particles in the PDMS solution.

Aggregation of the particles is largely influenced by the interparticle distance. Assuming a random dispersion of spherical particles, the interparticle distance, *H*, can be estimated from eqn (6):^19^6
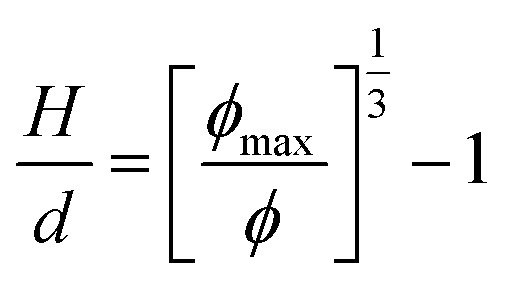
where *d* is the particle diameter, *ϕ*_max_ is the volume fraction at maximum random packing (≡0.64), and *ϕ* is the volume fraction of the particle. For 3 vol% of 5.1 μm iron particles, *H* is calculated to be 7.1 μm. Given the micron-scale interparticle gap, homogeneous random dispersion is expected with insignificant particle aggregation. Image analysis of the cross-section SEM micrographs (Fig. 2c) indicated 3.2% surface coverage of the PDMS matrix by the particles. If localized particle agglomeration occurred in the composites, the surface coverage from the cross-section SEM micrograph would deviate from 3 vol%. Hence, the surface coverage result suggests homogeneous dispersion of the iron particles in the PDMS matrices. For further quantitative analysis of the particle dispersity, the local connected fractal dimension was calculated by using the equations below:^20^

7 *M*(*ε*) ∝ *F*^*α*^_*ε*_

8
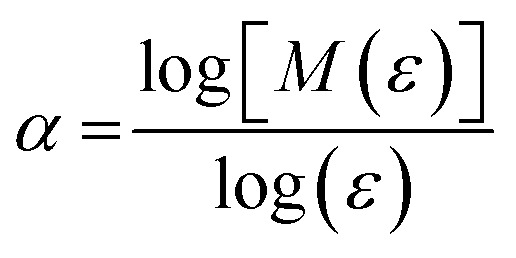
where *M*(*ε*) is the number of local connected pixel images in a *ε* sized box, *F* is the mass pre-factor, and *α* is the exponent characterizing the dimension. The two-dimensional binary image was utilized to estimate the local mass scaling properties. The *α* value can be calculated from *M*(*ε*) by increasing the box size (scale), *ε*. When the area of particle A is filled in matrix B, *α* = 2. When the portion of particle A in matrix B is a uniform straight line, *α* = 1, indicating one-dimensional random dispersion without aggregation; *α* values between 1 and 2 indicate local complexity as shown in the equation below:9
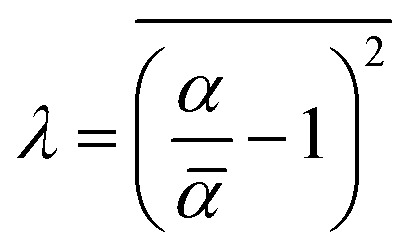
where *λ* is the lacunarity and the upper bar indicates the mean value. Note that *λ* = 0 when *α* and *ᾱ* are equal. The lacunarity is measure of the heterogeneity, *i.e.*, the discrepancy between the expected dimension of the mean value (*ᾱ*) of the local connected fractal dimension (*α*), indicating the gap between the patterns. Thus, a larger lacunarity represents larger gaps among patterns. From the binary SEM micrographs as shown in Fig. 2d, the local connected fractal dimension was calculated to be 1.22 with a lacunarity of 0.35. The fractal dimension close to unity and small lacunarity further substantiate the homogeneous dispersion of the iron particles in the PDMS matrices.

The synthesized 3D helical composite soft robots are shown in Fig. 2b. The helical geometry of the soft robots introduces rolling resistance, in contrast with the sliding resistance of the 2D square-shaped flat film. In addition, the helical soft robots experience significantly lower normal force during rolling, even when compared to cylinders with an identical radius of curvature, considering the equation below:^21^10
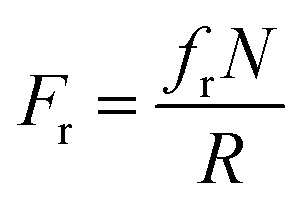
where *F*_r_ is the rolling resistance force, *f*_r_ is the rolling resistance coefficient, *N* is the normal force, and *R* is the radius of the cylinder or coil.

**Fig. 3:**
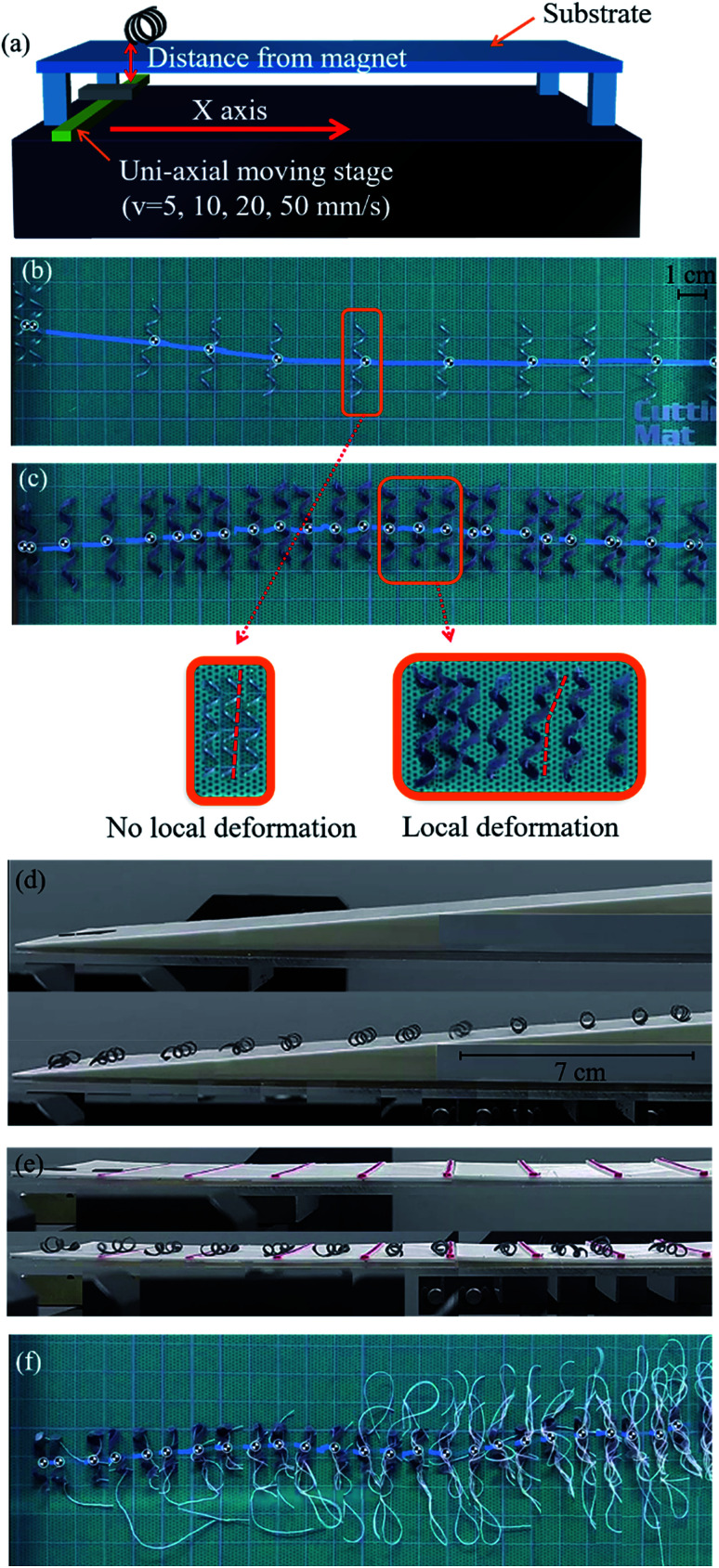
Magnetomotility achieved by uniaxial displacement of permanent magnet. (a) Scheme of the uniaxial moving stage set-up, (b) snap-shot images of rigid helical coil under magnetic conditions of 0.11 T and 10 mm s^−1^ with 1 s capture interval. (c) Snap-shot images of helical soft robots under magnetic conditions of 0.10 T and 10 mm s^−1^ with 1 s capture interval. (d) Magnetomotility of 2D flat film (top) and 3D helical soft robots (bottom) on 15° uphill slope (d), and discrete rectangular bars (e) with height increment from 0.2 mm to 2 mm. (f) Connection line- induced entanglement of tethered soft robots due to rolling motion.

The movements of the helical soft robots was regulated by uniaxial displacement of a NdFeB permanent magnet underneath the substrate. The position of the permanent magnet was controlled by a motorized linear translation stage (Fig. 3a). The magnitude of the magnetic flux density was determined by the distance between the magnet and the soft robots by simultaneous tracking of their dynamic positions.

The maneuverability of the helical soft robots was compared with that of the rigid analogue, made by alloy of chromium, aluminum and iron, having an identical helix diameter and length by capturing snap-shots at 3 s intervals, as shown in Fig. 3b and c. Despite the identical time interval for monitoring the movements, only a few images were acquired for the rigid coil in Fig. 3b, due to overlapping of the images when the coil remained in the stationary state for longer than the imaging time interval. Conversely, the soft robots in Fig. 3c had a significantly larger number of captured images without any overlapped images. This stark contrast originated from the mechanical stiffness of the rigid coil. In the case of the rigid coil, the rolling motion occurs only by global rotational motions without local coil deformation. The high zoom images clearly demonstrate various morphological and positional changes of the soft robots by local deformation, while the rigid coil only exhibited global rotational motions. The presence of local deformation modes also governed the threshold for actuation. The measured threshold for the rigid coil was 0.11 T, which is drastically larger than that of the soft robots (0.05 T). Note that the body-weight of the soft robots was larger than that of the rigid analogues, providing a larger normal force in the rolling resistance. Hence, the efficient rolling of the soft robots was again attributed to the local deformation, leading to the low threshold. Notably, the local deformation of the soft robots allowed more continuous motility with reduced residence time in stationary positions. In the case of the rigid coil, the rolling motion was rather irregular and the coil resided in the stationary position for longer periods (Fig. 3b). Conversely, the soft robots demonstrated smoother rolling behavior with significantly reduced dwell time due to the local deformation (Fig. 3c).

Time-resolved monitoring of the 3D helical soft robots was carried out to elucidate the effects of the local deformation and rolling resistance. In the case of the 2D squared flat film with sliding resistance, the threshold for movement was measured to be 0.11 T. Conversely, that of the helical soft robots was only 0.05 T, despite the identical composite mass and chemical composition due to the introduction of rolling resistance, clearly demonstrating the efficiency of the design of the 3D helical soft robots. The movements of the 2D flat film and 3D helical soft coil were compared on a 15° uphill slope (Fig. 3d) and discrete walls (Fig. 3e). The displacement of the 2D flat film was only 2.27 cm up to the point of cessation of the motility, corresponding to a height of 0.58 cm and magnetic flux density of 0.11 T. In comparison, the displacement of the 3D helical soft robots reached 19.45 cm, corresponding to a height of 5.03 cm and magnetic flux density of 0.01 T. The magnetomotility of soft robots at low magnetic flux density is essential for operation from a distance when the thickness of the substrate is substantial. In addition, a load-bearing system can be achieved at the expense of the magnetic threshold and kinetic energy.

As shown in Fig. 3e, the movements of the 2D film and 3D helical soft robots were contrasted on discrete walls ranging from 0.2 mm to 1.6 mm in height with a height increment of 0.2 mm. The 3D helical soft robots overcame the obstacles up to 1.4 mm, whereas the 2D film stopped at 0.2 mm bar. This comparison proves that the construction of a 3D helical geometry is an effective strategy for designing soft robots that can overcome obstacles and to navigate through rough terrains.

To date, a large portion of soft robots are operated by tethered systems such as electrically actuated caterpillars,^2^ pneumatically actuated artificial tentacles,^3^ and elastomeric walkers.^4^ These soft robots do not suffer from entanglement of the connection lines due to the absence of rotational motility. However, in the rolling motion of tethered systems, as shown in Fig. 3f, the rotational motility induces entanglement of the connection lines. From comparison of Fig. 3f with Fig. 3c, it is clear that the untethered systems are advantageous for achieving rotational motion without interference from the connection line.

**Fig. 4:**
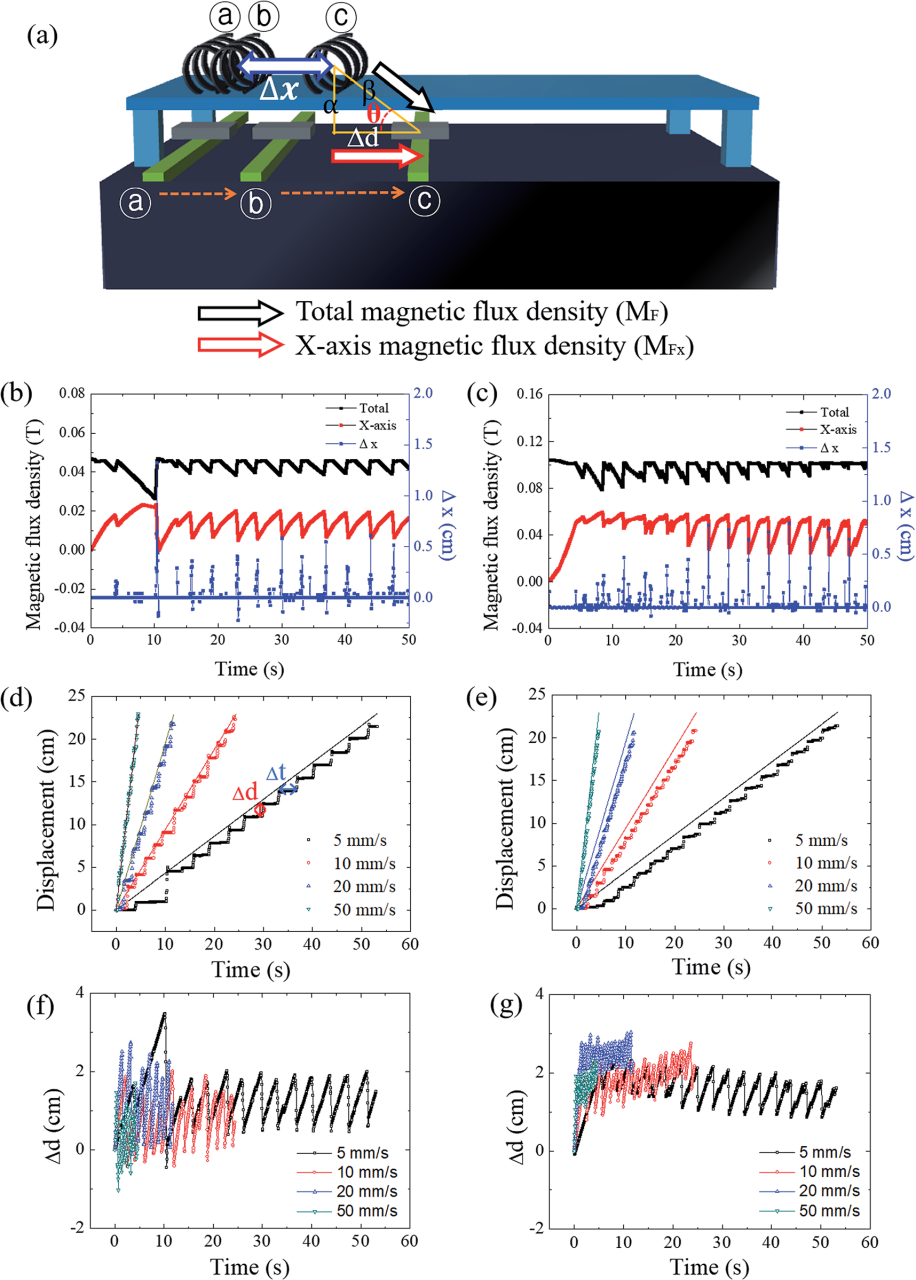
Time-resolved magnetomotility analysis of the 3D helical soft robots with variation of the magnetic flux density and magnet velocity conditions. (a) Scheme for deconvolution of magnetic flux density into *x* and *y* components. (b) Correlation between simulated magnetic flux density and soft robots displacement under initial magnetic flux density of 0.05 T (b) and 0.10 T (c). Time-resolved displacement of soft robots with variation of the magnet velocity under initial magnetic flux density of 0.05 T (d) and 0.10 T (e). Time-resolved spatial deviation of soft robots with variation of the magnet velocity under initial magnetic flux density of 0.05 T (f) and 0.10 T (g).

Fig. 4a shows the deconvolution of the total magnetic flux density into the *x* and *y* components. The distance between the soft robots and the magnet (the *x*-axis spatial deviation (Δ*d*)) was measured from the horizontal axis. The vertical distance between the soft robots and the magnet (*α*) was utilized for calculation of the angle (*θ*), along with Δ*d*. The *x*-component of the magnetic flux density was calculated by using the diagonal distance (*β*) using the equations given below:11
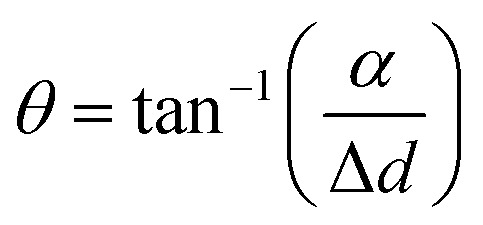
12
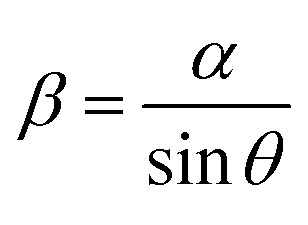

13 *M*_F*x*_ = *M*_F_ cos *θ*

where the *M*_F*x*_ is the *x*-component of the magnetic flux density, and *M*_F_ is the total magnetic flux density.

The time-resolved magnetomotility was analyzed under initial magnetic flux densities of 0.05 T and 0.10 T with variation of the magnet velocity (Fig. 4b–e). The positions of the soft robots and the magnet were determined from their center of mass. Local deformation led to some variance in the *x*-axis magnetic flux density. Rolling motility of soft robots is activated when the magnetically induced momentum exceeds the rolling resistance and inertia. Hence, the total magnetic flux density does not directly govern the movements, and the *x*-axis magnetic flux density is more influential. When the *x*-position of the magnet and the soft robots are aligned, the magnetic field is vertically aligned and the *y*-component of the magnetic flux density is equal to the total magnetic flux density. At a constant magnet velocity of 5 mm s^−1^, there is spatial deviation between the soft robots and the magnet (Δ*d*), which increases the distance between the magnet and the soft robots (*β*). Here, the *x*-axis magnetic flux density increases despite the decrease in the total magnetic flux density, indicating that rolling is governed by the magnitude of the *x*-axis magnetic flux density of this device, rather than by the total magnetic flux density. As evident from Fig. 4c, however, rolling was not always observed at the maximum *x*-axis magnetic flux density, as rolling motility is the consequence of the interplay of various parameters. When the accumulated magneto-strain and the gravitational force outweigh the rolling resistance and inertia, sudden displacement of the soft robots would be initiated by the rolling motion. The rolling motion of the helical soft robots decreases *β*, again increasing the total magnetic flux density. Once the soft robots overcame the initial inertia, relatively uniform but intermittent magnetomotility was observed from *ca.* 10 s. For an initial magnetic flux density of 0.10 T (Fig. 4c), the *x*-axis magnetic flux density became larger with reduced fluctuations, revealing the effects of the magnetic flux density on inertia.

The temporal and spatial deviation of the soft robots were quantified at magnet velocities of 5, 10, 20, and 50 mm s^−1^ under initial magnetic flux densities of 0.05 T and 0.10 T, as shown in Fig. 4d and e, respectively. The aforementioned initial delay of 10 s (Fig. 4d) was observed due to the force of inertia that acts opposite to the direction of motion under the influence of frictional force. The spatial deviation of the soft robots from the magnet can be defined by:

14 Δ*d* = *d*_m_ − *d*_s_

where *d*_m_ is the displacement of the magnet and *d*_s_ is the displacement of the soft robots at a certain time. Similarly, the temporal deviation (Δ*t*) can also be calculated by:

15 Δ*t* = *t*_s_ − *t*_m_

where *t*_m_ is the time required for the magnet to reach a certain *x*-position and *t*_s_ is the time required for the soft robots to reach the targeted location. The spatial and temporal deviations both oscillate due to the intermittent magnetomotility. In general, suppression of the intermittent behavior was achieved with a larger magnetic flux density and faster magnet velocity due to enhancement of the forces acting in the direction opposite to the force of inertia. Fig. 4f and g demonstrate the oscillatory spatial deviation under initial magnetic flux densities of 0.05 T and 0.10 T, respectively. Reduced fluctuation of the spatial deviation was observed with a stronger magnetic flux density as evident in Fig. 4g. Unlike the case with the initial magnetic flux density of 0.05 T, the spatial deviation was positive for the entire region in the case of the initial magnetic flux density of 0.10 T. The temporal deviations followed a trend similar to that of the spatial deviation (see ESI, Fig. S3a and b†).

**Fig. 5:**
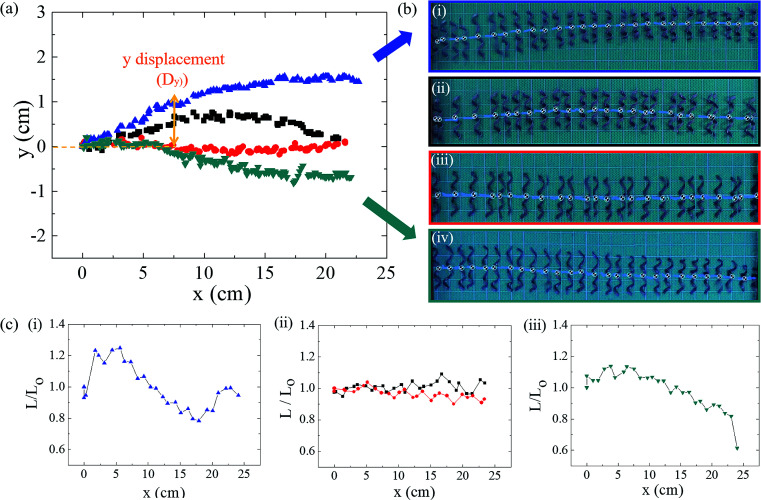
Displacement on *y*-axis and relative length of soft robots under various magnetic conditions. (a) *x*–*y* displacement of the soft robots, (b) trajectory images of the soft robots, (c) relative length against *x*-axis of the soft robots. Blue triangles (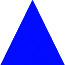) and (i) indicate right-handed soft robots under initial magnetic flux density of 0.15 T and magnet velocity of 20 mm s^−1^. Black squares (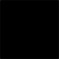) and (ii) indicate right-handed helical soft robots under initial magnetic flux density of 0.10 T and magnet velocity of 10 mm s^−1^. Red circles (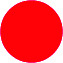) and (iii) indicate left-handed soft robots under initial magnetic flux density of 0.10 T and magnet velocity of 10 mm s^−1^. Cyan inverted triangles (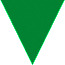) and (iv) indicate left-handed soft robots under initial magnetic flux density of 0.15 T and magnet velocity of 20 mm s^−1^.

Helical soft robots are intrinsically chiral. To elucidate the effects of the chirality on the magnetic movements, the trajectories of the left-handed and right-handed soft robots are compared in Fig. 5a. At a magnetic flux density of 0.15 T and magnet velocity of 20 mm s^−1^, the *y*-position of the right-handed soft robots (blue triangle) deviated positively. Opposite to this upward deviation, the left-handed soft robots moved in the downward direction, while the target position (magnet location) varied solely on the *x*-axis. The helical geometry induces deviation of the *y*-position due to the directionality of the translational motility orthogonal to the rotational axis.^6^ Thus, the directionality of the *y*-positional deviation is determined by the helix handedness.

At a glance, it may seem that the *y*-positional deviation can be prevented by increasing the magnetic flux density with a larger attractive force. However, greater inertia also acts in the rolling direction. The optimal condition for suppression of the *y*-positional deviation is found from the 0.10 T magnetic flux density and 10 mm s^−1^ for both the right-handed and left-handed soft robots. In addition to the positional deviations, spring-like soft robots undergo dimensional deviations such as changes in length due to deformation during rolling (Fig. 5c). The relative length is defined by:16
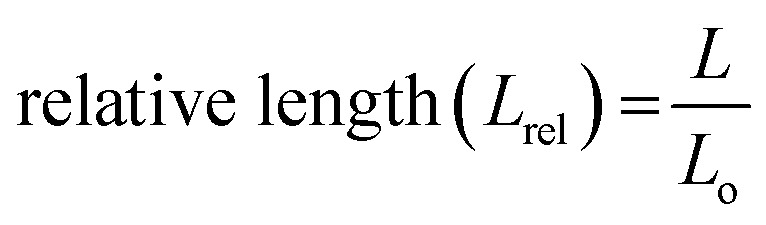
where the *L* is the deformed length of the soft robots during the rolling motion and *L*_o_ is the initial length of the soft robots in the absence of a magnetic field. Significant dimensional fluctuations were observed under magnetic conditions of 0.15 T and 20 mm s^−1^, where *L*_rel_ = 0.78–1.24 for the right-handed soft robots and *L*_rel_ = 0.61–1.14 for the left-handed soft robots. In addition to the suppressed *y*-positional deviations under conditions of 0.10 T and 10 mm s^−1^, reduced variation of the relative length (*L*_rel_) was achieved regardless of the handedness (*L*_rel_ = 0.95–1.09 for right-handed soft robots and *L*_rel_ = 0.90–1.04 for left-handed soft robots).

**Fig. 6:**
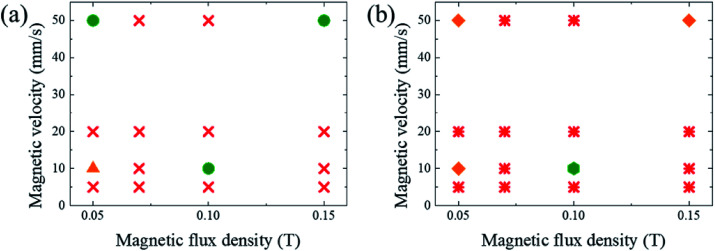
Phase diagrams for optimal conditions with lowest deviation. (a) Phase diagram for the average spatial deviation on *y*-axis with respect to helical diameter, *D*_h_. Green circles (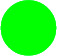):
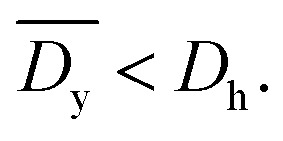
 Orange triangles (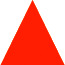): 
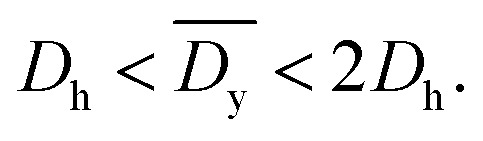
 Red *×* (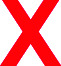): 
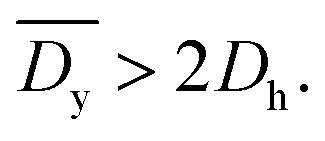
 (b) Phase diagram for average temporal deviation with respect to time for displacement of the helix diameter, *t*_D_. Green hexagons (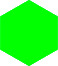): 
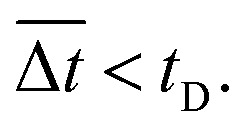
 Orange diamonds (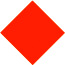): 
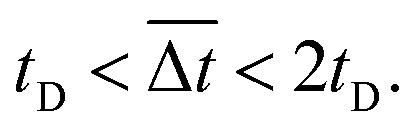
 Red asterisks (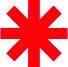):
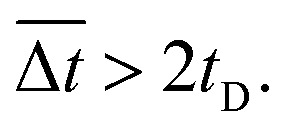

It was demonstrated that the magnetomotility of helical soft robots can be regulated by manipulating the magnetic flux density and the magnet velocity. To scrutinize and optimize the magnetomotility of the soft robots, phase diagrams of the spatial and temporal deviations under various magnetic conditions were constructed, as shown in Fig. 6. The spatial deviation (in terms of the *y*-position) and temporal deviation were normalized with respect to the helical diameter (*D*_h_ = 4.5 mm). The spatial deviation is divided into three regimes: (1) 
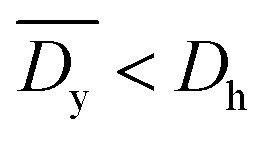
 (green circles, 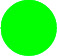), (2) 
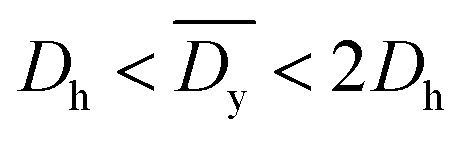
 (orange triangles, 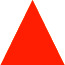), and (3) 
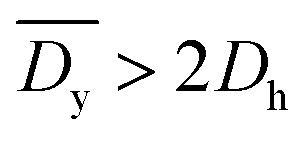
 (red *×*, 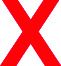). The average temporal deviation may serve as a measure of the responsivity of the soft robots to the applied magnetic field. The temporal deviation is also divided into three regimes: (1) 
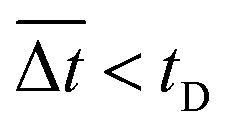
 (green hexagons, 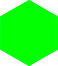), (2) 
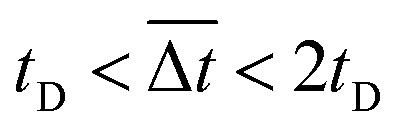
 (orange diamonds, 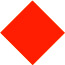), and (3) 
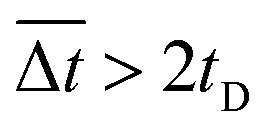
 (red asterisks, 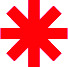). For example, at 10 mm s^−1^, if 
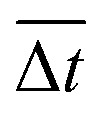
 is smaller than 0.45 s, it falls under the regime denoted by the green hexagons (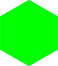), if 
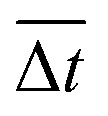
 is between 0.45 s and 0.9 s, it falls under the regime denoted by the orange diamonds (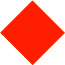), and if 
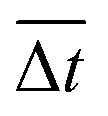
 is larger than 0.9 s, it falls under the regime denoted by the red asterisks (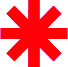). Based on the spatial and temporal phase diagrams, successful untethered maneuver of the helical soft robots was achieved using magnetic conditions of 10 mm s^−1^ and 0.10 T. Therefore, we demonstrated linear maneuver of helical soft robots regardless of the handedness while maintaining facile motility at low magnetic flux density *via* introduction of rolling resistance.

## Conclusions

In conclusion, untethered manipulation of a 3D helical soft robots was achieved at ambient temperature by accomplishing magnetomotility without any electrical circuits, complicated algorithms, or connection lines. The magnetically active soft robots were prepared by dispersing 5.1 μm iron particles in a PDMS matrix. Random particle distribution was achieved in the highly viscous PDMS solution, as scrutinized by the image analysis of the SEM micrographs and local connected fractal dimensional analysis. The 3D helical soft robots required less energy for maneuver due to the introduction of rolling resistance instead of the sliding resistance that is operative in the 2D square-shaped soft film. Hence, the 3D helical soft robots were capable of overcoming obstacles and could move uphill at a lower magnetic threshold than that of the 2D composite film. In comparison with the rigid coil that repeatedly showed irregular cessation of rolling, the soft robots demonstrated a steady rolling motion with intermittent local deformation, which reduced the threshold magnetic flux density for deformation. Despite the efficient maneuver of the helical soft robots, the intermittent rolling motility resulted in spatial and temporal deviations between the magnet and soft robots. Further, the helix angle and handedness generated upward or downward deviation on the *y*-axis. By mapping the phase diagram for both spatial and temporal deviation based on the magnetic flux density and magnet velocity, the optimal conditions for maneuvering the robot were achieved with a magnet velocity of 10 mm s^−1^ and magnetic flux density of 0.10 T. Therefore, efficient linear magnetomotility of 3D helical soft robots was effectively accomplished by suppressing the spatial (*x*- and *y*-positional) and temporal deviations.

## Conflicts of interest

There are no conflicts to declare.

## Supplementary Material

RA-009-C9RA01775E-s001

RA-009-C9RA01775E-s002

RA-009-C9RA01775E-s003

RA-009-C9RA01775E-s004

RA-009-C9RA01775E-s005

RA-009-C9RA01775E-s006
