# Supplementary material for: Magnetomotility of untethered helical soft robots
Source: RSC Adv. 2019 Apr 10;9(20):11272–80. doi: 10.1039/c9ra01775e (PMC9062993; doi:10.1039/c9ra01775e)
Supplement: RA-009-C9RA01775E-s006 [file RA-009-C9RA01775E-s006.pdf]

Electronic Supplementary Material (ESI) for RSC Advances.
This journal is © The Royal Society of Chemistry 2019

SUPPLEMENTARY INFORMATION

Magnetomotility of Untethered Helical Soft Robots

Jeong Eun Park,[1] Jisoo Jeon,[1] Jae Han Cho,[1] Su Kyoung Won,[1] Hyoung-Joon Jin,[1,2] Kwang Hee Lee,[1] and Jeong Jae Wie[*,1,2]

[1]Department of Polymer Science and Engineering, Inha University, Republic of Korea

[2]World Class Smart Laboratory (WCSL), Inha University, Republic of Korea

Quadratic function of terminal velocity according to radius of the iron particles; digital images of several samples compared to those of the soft robots; time-resolved temporal deviation of soft robots; measured velocity of linear translational stage for the magnet; Movie 1 contrasts global rotational motility of rigid helical coil and local deformation of helical robot; Movie 2 shows efficient climbing movements of 3D helical soft robots on 15° uphill slope by exploiting the rolling resistance, unlike the inefficient movements of the square shaped 2D film operating via sliding resistance; Movie 3 shows 3D helical soft robots rolling over walls (wall height was increased in 0.2 mm increments) in contrast with the cessation of the dragging motion by the square-shaped 2D film; Movie 4 demonstrates a slight deviation from linear trajectory from the backward rolling in comparison with forward rolling despite the identical magnetic conditions (0.10 T, 10 mm/s). This is attributed to the chirality of helix since the magnetic condition is optimized for forward rolling.

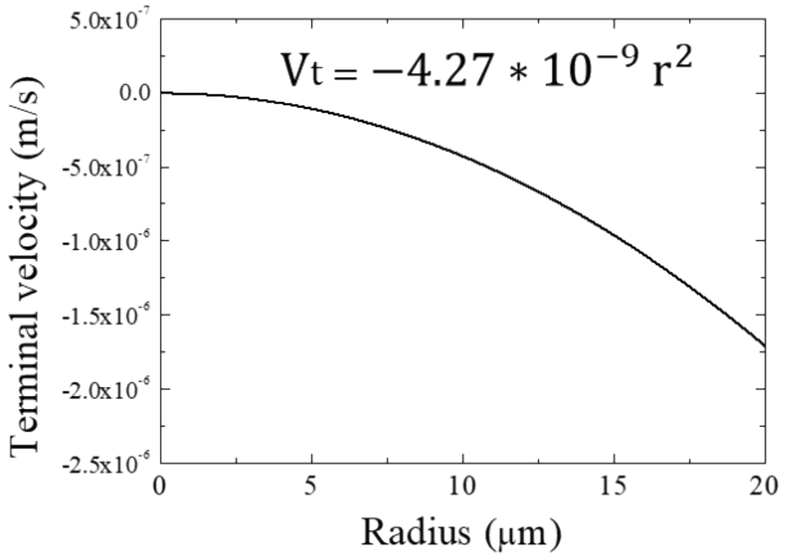

Figure S1. Quadratic function of terminal velocity according to radius of the iron particles. To demonstrate the dispersity of the iron particles in the PDMS matrices, particle analysis can be used to indicate the force balance.

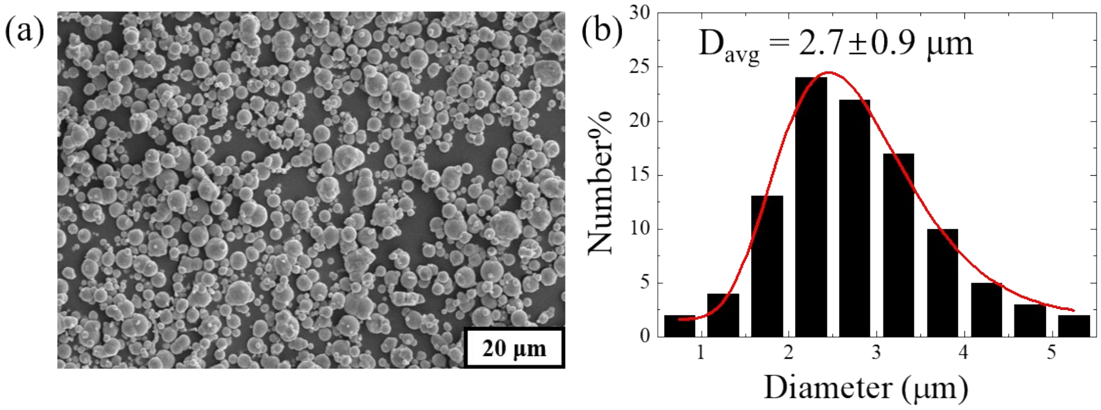

Figure S2. (a) SEM micrograph of iron particles (b) Histogram for diameter of iron particles based on the image analysis from the SEM micrograph in Figure S2(a).

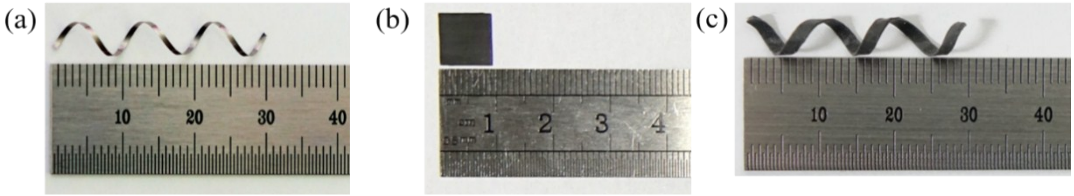

Figure S3. Digital images of several samples compared to the soft robots. (a) Helical coil from rigid alloy (Cr 20%/Al 3%/Fe 77%) having identical helix angle, length, and handedness as the helical soft robots. (b) Square PDMS-CIP composite 2D film (95 mm (L) × 95 mm (W) × 0.4 mm (T)) with weight (40 mg) identical to that of the 3D helical soft robots. (c) Left-handed helical soft robots with helix angle and length identical to that of the right-handed helical soft robots.

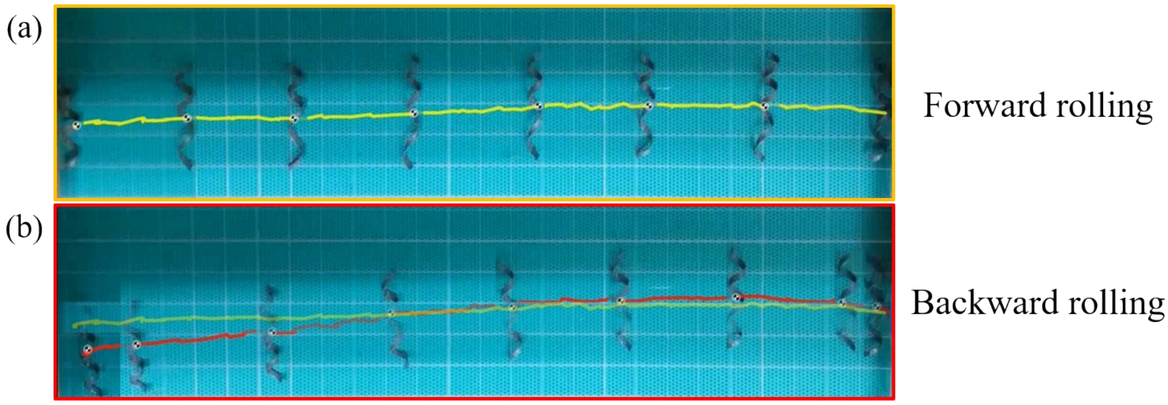

Figure S4. Snapshot of trajectory for forward and backward rolling of helical soft robots (a) Forward and (b) backward rolling under the same magnetic conditions (magnetic velocity of 10 mm/s and magnetic flux density of 0.10 T). Time interval of snapshots was 4 s. The yellow and red line indicates trajectories for forward and backward rolling, respectively. Unlike the linear trajectories observed in the forward rolling, the backward rolling was slightly deviated from the original trajectory due to chiral characteristics.

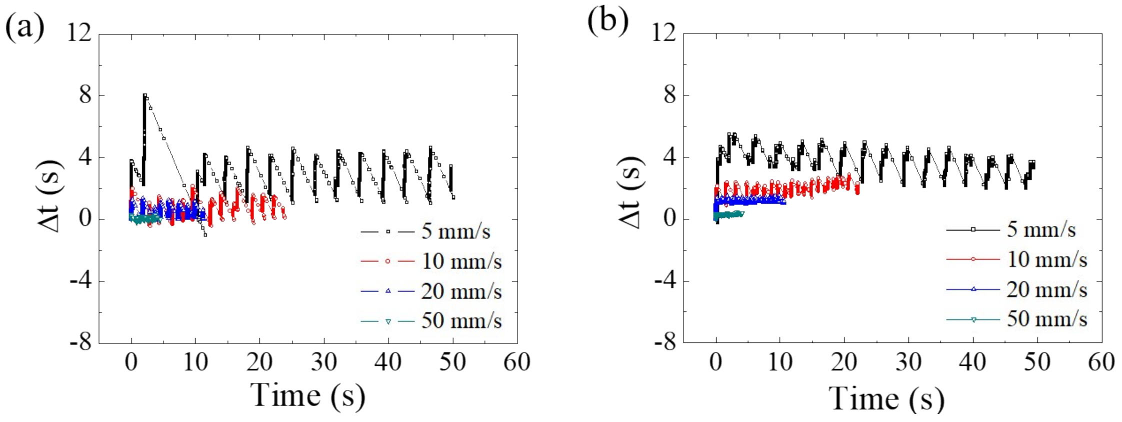

Figure S5. Time-resolved temporal deviation of soft robots with variation of the magnet velocity conditions. Initial magnetic flux density of 0.05 T (a) and 0.10 T (b); temporal deviation (Δt) between magnet and soft robots was measured. The magnitude of deviation at 0.10 T was positive for the entire time region and the fluctuations were smaller than that at 0.05 T due to the lower force of inertia in the opposite direction.

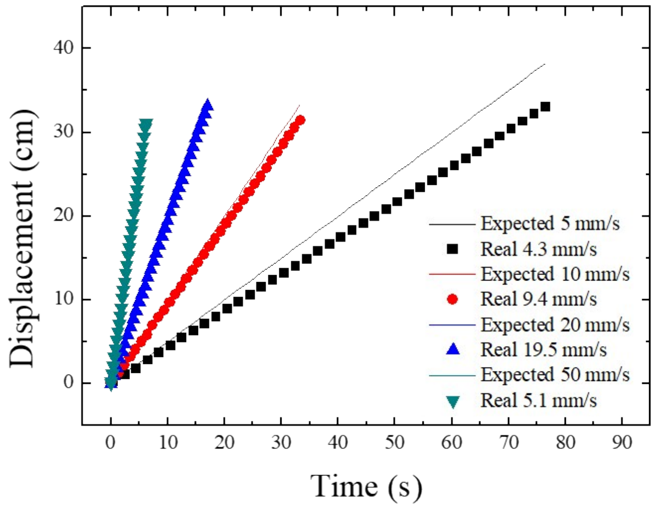

Figure S6. Measured velocity of linear translational stage for the magnet. The nominal velocity conditions utilized in the experiment differ from the values measured by using a high-speed camera. The velocity was calibrated against the measured value and then utilized in the analysis.
